# An Experience of the Ibaraki Disaster Psychiatric Assistance Team on the Diamond Princess Cruise Ship: Mental Health Issues Induced by COVID-19

**DOI:** 10.1017/dmp.2020.305

**Published:** 2020-08-12

**Authors:** Sho Takahashi, Kazunori Manaka, Takafumi Hori, Tetsuaki Arai, Hirokazu Tachikawa

**Affiliations:** Department of Disaster and Community Psychiatry, Faculty of Medicine, University of Tsukuba, Ibaraki, Japan; Ibaraki Prefectural Medical Center of Psychiatry, Kasama, Ibaraki, Japan; Department of Psychiatry, Faculty of Medicine, University of Tsukuba, Ibaraki, Japan

**Keywords:** COVID-19, diamond princess, quarantine, mental health

On February 3, 2020, the Diamond Princess cruise ship arrived at Yokohama Port, Japan. The ship was locked down by the Japanese Government because 1 passenger was found to be infected by coronavirus disease (COVID-19). Since February 5, the ship was quarantined for 14 days with passengers from 52 countries required to stay in their own cabins. During that period, the number of infected cases steadily increased. Finally, 696 of the 3618 persons on board were found to test positive.^1^ Although there was a chronological review from newspapers^2^ and the patient transportation missions^3^ were reported, there have been no reports on the mental health of the passengers. Here we report on the disaster mental health services on the Diamond Princess.

To respond to the mental health needs on the ship and by requests of the Japanese Disaster Psychiatric Assistance Team (DPAT) secretariat, we, along with other medical support teams, boarded the ship and provided mental health services as the Ibaraki DPATs^4^ from February 11 to 12, that is, in the midst of the quarantine period. Psychiatrists and nurses visited passengers in their rooms with personal protective equipment whenever requested. We examined 14 passengers among the 20 requests received. The complaints varied, including anxiety, panic, insomnia, fatigue, depression, anger, and suicidal ideation. All case patients were worried about infection and felt anxiety about isolation and separation. For example, 1 man was forced to disembark because of infection by COVID-19, and his wife, left alone in the cabin, felt hopeless and suicidal. All aboard claimed insufficient interactive communication because all room telephone lines were always occupied. The mental state of most clients was improved by brief counseling, but 6 clients needed psychotropic medications. Some of the crew and medical staff on the ship had feelings of exhaustion, emotional instability, and insomnia. They had to continue servicing the passengers, despite their own fears of infection. We supported crews and medical workers as well as the passengers, as in the cases of previous disasters. The medical teams, Japanese Government, and crew did their best and, finally, completed the mission. However, we also had to isolate ourselves for more than 14 days after the mission, despite testing negative, due to the strict rules for close contact persons. We felt the same fear and anger that the passengers experienced.

The quarantine process induced significant psychological distress in every relevant person because of the personal isolation and fear of personal safety as well as the fear of safety for their loved ones.^5^ To reduce the mental health problems in such cases, disaster mental health services like DPATs are available to prevent worsening and such severe conditions as suicide attempts and mass panic. Crews are key persons in solving these difficult situations, so mental health support for them is especially important. Separation and isolation of family passengers should be avoided. Interactive risk communication tools for multi-language and various ages should be developed. This is the important lesson from our experience on the Diamond Princess.
